# Neutrophil/lymphocyte and monocyte/lymphocyte indexes as potential predictors of relapse at 1 year after diagnosis of pediatric multiple sclerosis: a single-center, exploratory and proof-of-concept study

**DOI:** 10.3389/fnins.2023.1305176

**Published:** 2024-01-15

**Authors:** Filipe Palavra, Leonor Geria, André Jorge, Margarida Marques, Constança Soares dos Santos, Joana Amaral, Joana Afonso Ribeiro, Cristina Pereira, Conceição Robalo

**Affiliations:** ^1^Center for Child Development–Neuropediatrics Unit, Hospital Pediátrico, Centro Hospitalar e Universitário de Coimbra, Coimbra, Portugal; ^2^Laboratory of Pharmacology and Experimental Therapeutics, Faculty of Medicine, Coimbra Institute for Clinical and Biomedical Research (iCBR), University of Coimbra, Coimbra, Portugal; ^3^Clinical Academic Center of Coimbra, Coimbra, Portugal; ^4^Faculty of Medicine, University of Coimbra, Coimbra, Portugal; ^5^Department of Neurology, Centro Hospitalar e Universitário de Coimbra, Coimbra, Portugal; ^6^Biostatistics and Medical Informatics Laboratory, Faculty of Medicine, University of Coimbra, Coimbra, Portugal

**Keywords:** multiple sclerosis, children, adolescents, neutrophil-lymphocyte index, monocyte-lymphocyte index

## Abstract

**Introduction:**

Early identification of patients with a more unfavorable outcome in Multiple Sclerosis (MS) is crucial to optimize individualized treatment. Neutrophil-lymphocyte index (NLI) and monocyte-lymphocyte index (MLI) have been considered as potential biomarkers for disease prognosis. Our study aims to investigate the usefulness of NLI and MLI as predictors of relapse, disability progression, and lesion accumulation on magnetic resonance imaging (MRI) 1 year after diagnosis and treatment initiation, in pediatric-onset MS.

**Methods:**

A retrospective single-center study was conducted, including patients with diagnosis of MS established in pediatric age (<18 years old), at least 1-year of follow-up, and a complete blood count (CBC) performed at diagnosis. We collected the nearest-to-diagnosis NLI and MLI, as well as clinical and imaging variables, at diagnosis and 12 months later. Our cohort was further dichotomized into two groups, based on the presence of relapses. Statistical significance was considered for *p* < 0.05.

**Results:**

Eighteen patients (*n* = 18) were included. The relapsing group had higher mean, minimum, and maximum values for both NLI (5.17 ± 5.85, range: 1.57–11.92) and MLI (0.35 ± 0.22, range: 0.19–0.59), compared to the non-relapsing group (2.19 ± 1.63, range: 1.12–7.32 for NLI, and 0.24 ± 0.09, range: 0.14–0.44 for MLI). A higher percentage of patients in the relapsing group had increased NLI (>1.89, 66.7%) and MLI (>0.21, 66.7%) values than those in the non-relapsing group (46.7%). Patients who presented new T2-hyperintense lesions on MRI after 1 year of follow-up also had higher mean, minimum, and maximum values of both biomarkers. Patients who did not achieve No Evidence of Disease Activity-3 (NEDA-3) state exhibited higher values for both ratios. However, in our sample, no statistically significant correlations were found between MLI and NLI values and the clinical and imaging variables considered.

**Conclusion:**

The ease of obtaining NLI and MLI from routine blood tests renders them useful biomarkers as a screening tool in longitudinal follow-up. Our study was based on a very small sample size, but it allowed us to verify the feasibility of the protocol used. It is intended to involve other centers in the next phase of this work, testing the possible usefulness of the indices under analysis on a larger sample.

## 1 Introduction

Multiple sclerosis (MS) is a chronic immune-mediated, inflammatory, and demyelinating disease affecting the central nervous system (CNS). It mainly develops during early adulthood, with the majority of diagnoses being established between 20 and 40 years of age (Confavreux et al., 1980). Although pediatric MS is considered a rare condition, between 3–5% of patients have an onset of disease under the age of 18 (Boiko et al., 2002; Trabatti et al., 2016; Thompson et al., 2018). Younger children often present with polyfocal symptoms, while adolescents mainly present with single focal deficits, as is often seen in adults (optic neuritis, myelitis and brainstem syndromes are also typical manifestations in this age group) (Mikaeloff et al., 2004). Neurodegeneration also occurs at this early stage of life and frequently translates into sensory, motor and brainstem dysfunction, in clinical practice (Banwell et al., 2007). Moreover, cognitive disability has become a topic of great clinical relevance, being reported in approximately 30% of pediatric MS patients (Parrish et al., 2014). When compared with adult MS population, children are at a higher risk of impaired vocabulary and language-based cognition (Parrish et al., 2014). A solid association between cognitive impairment and EDSS (Expanded Disability Status Scale) score, number of relapses, and disease duration has been demonstrated, based on the neuropsychological assessment to which pediatric MS patients are submitted (MacAllister et al., 2005).

In 95–98% of pediatric MS patients, the clinical course is relapsing-remitting (RRMS) (Renoux et al., 2007). Children tend to have a high relapse rate, especially in the first years following the diagnosis and studies have reported 2.3–2.8 times higher relapse rates in pediatric MS patients, showing that, in its earliest stages, the disease has a more important inflammatory component than when it only starts in adulthood (Gorman et al., 2009; Narula, 2016). This is also true considering MRI outcome measures (lesion load/active MS lesions): it has been reported that children present a higher number of T2-hyperintense lesions than adults, which are larger and more ill-defined (Waubant et al., 2009). Nevertheless, several studies demonstrated a limited correlation between clinical (relapses/disability) and imaging outcome measures (data dealing with inflammatory imaging markers), in terms of MS disease progression. These results tend to reflect routine practice and validate the concept of clinico-radiological dissociation of disease activity (van Faals et al., 2020).

Being, therefore, a disease in which inflammation assumes such a relevant nature, at least in its earliest stages, it has become clinically pertinent to search for biomarkers that, precisely involving inflammation, can be used as possible predictors of the clinical and radiological activity of the disease, as well as of its eventual progression. Both neutrophils and monocytes have been linked to pro-inflammatory activity in MS, making part of the chronic systemic inflammatory environment that characterizes the disease, despite lymphocytes’ major relevance. NLI (neutrophil-lymphocyte index, which characterizes the balance between neutrophil and lymphocyte levels) and MLI (monocyte-lymphocyte index, which characterizes, in the same way, the balance between monocyte and lymphocyte levels) have been described as useful biomarkers for disease prognosis, by tracking the relative changes in longitudinal follow-up of the patients (Martins et al., 2011). Even so, an increase in these indices (whether due to an increase in the neutrophil or monocyte count or even a relative reduction in the lymphocyte number) can translate into an immune imbalance in favor of the innate response itself (more dependent on neutrophils and monocytes, which can even suppress lymphocyte activity), to the detriment of the adaptive response, in which lymphocytes play a fundamental role. Now, at the beginning of a chronic disease such as MS, the existence of an imbalance of this type could hypothetically have a pathophysiological significance, which will need to be explored (Martins et al., 2011; Uysal et al., 2014). Several prognostic indicators of MS relapses have been identified, including MRI, serum neurofilament light chains, the EDSS score, and the relapse rate within the first year of diagnosis. However, MRI is associated with significant cost, while the test for serum neurofilament light chains remains limited in its availability. In contrast, NLI and MLI can be quickly and cheaply obtained from the complete blood count (CBC) that is routinely performed on patients, at the time of the diagnosis. Calculating these ratios is simpler than measuring the levels of other inflammatory cytokines, such as tumor necrosis factor (TNF), interleukin-6 (IL-6), and interleukin-1β (IL-1β) (Martins et al., 2011; Uysal et al., 2014). A recent study showed evidence that elevated NLI and MLI correlated with higher rate of relapse and increased MS-related disability (Huang et al., 2022).

With this study, we aimed to investigate the usefulness of NLI and MLI biomarkers as predictors of relapses, disability progression (as measured by the EDSS score), and accumulation of lesions in the MRI, 1 year after diagnosis and targeted treatment of pediatric-onset MS patients. By testing our analysis protocol on a small sample of patients, we also intend to evaluate its practical feasibility, with a view to subsequently carrying out a larger, multicenter study.

## 2 Materials and methods

### 2.1 Study design and data source

We conducted a retrospective observational and proof-of-concept study using data from our research database (Hospital Pediátrico, Centro Hospitalar e Universitário de Coimbra). This study was approved by the local Ethics Committee (reference PIOBS.SF.138-2022). All participants (or caregivers) gave their consent to participate in the study. The study followed the principles of the Declaration of Helsinki, national legislation for clinical research and good clinical practice principles (ICH-GCP).

### 2.2 Study population

We included patients with a confirmed diagnosis of MS established in pediatric age (less than 18 years old), according to the 2012 IPMSSG and the revised 2010 McDonald diagnostic criteria, observed at the Demyelinating Disorders Consultation of our center (diagnoses were made between the years 2000 and 2021). The analyses included a 1-year follow-up period, measured from the first record point, that was defined as the time of the diagnosis. It was also required for enrollment to have carried out a CBC at the time of the diagnosis. Considering pediatric MS a very inflammatory disease, as we know it is, it was our intention, when establishing this short follow-up (of only 1 year), to try to verify whether the ratios were related to early activity of the disease, one of the main focuses of research in our group.

**FIGURE 1:**
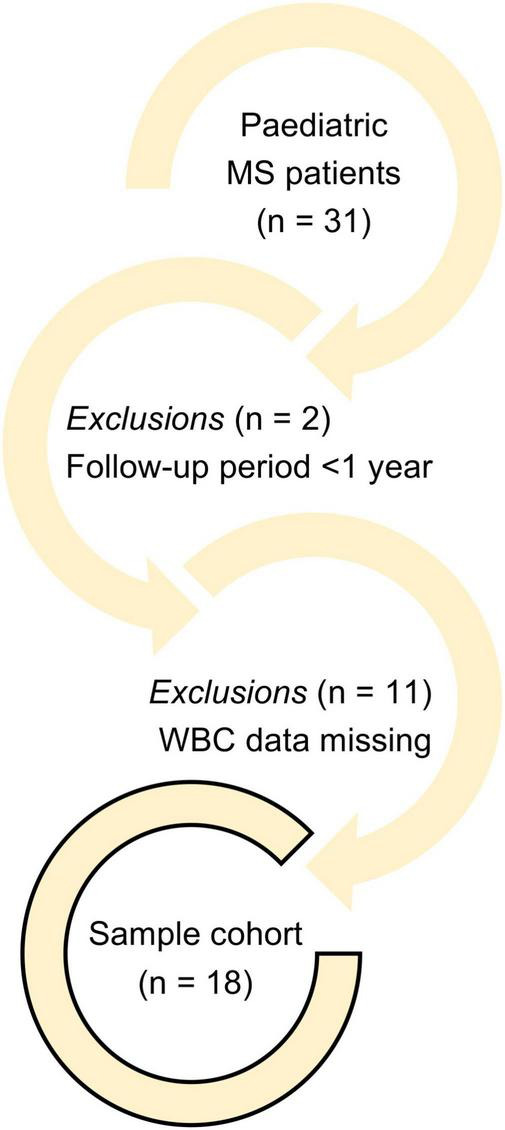
Enrolment strategy of pediatric MS patients.

We excluded patients who have not yet had a follow-up period of at least 1 year. Likewise, we did not consider patients without white blood cells (WBC) data within 90 days of the first record point. Figure 1 represents the enrolment strategy of patients. A total of 18 MS patients (3 with relapses during the first year of follow-up and 15 without) were included in the final analyses.

### 2.3 Variables analyzed

Patient demographic characteristics, including age at MS diagnosis and sex were collected. Clinical data such as MS phenotype, EDSS score at diagnosis, number of relapses previously to MS diagnosis, topography of MS lesions from previous relapses and prescribed disease modifying therapy (DMT) at diagnosis were registered. Additionally, 12 months after the diagnosis, we collected the number of relapses and the EDSS score, as well as the fulfilment of the NEDA-3 state (no evidence of relapses, no disease progression, and no new MRI activity).

The fulfillment of the revised 2010 McDonald diagnostic criteria, with evidence of dissemination in time and space, the number of T2-hyperintense lesions and gadolinium-enhancing lesions on MRI, at the time of the diagnosis, were recorded. One year after the diagnosis, we ascertained the number of T2-hyperintense lesions and gadolinium-enhancing lesions on MRI. The 2010 McDonald criteria were considered, as several of the patients included in the cohort were diagnosed based on them. All patients meeting the 2017 McDonald criteria also met the 2010 McDonald criteria.

In terms of laboratory variables, NLI and MLI closest to the first record point were acquired. No study of lymphocyte subpopulations was conducted.

### 2.4 Statistical analyses

We performed a descriptive analysis of the demographic characteristics, clinical and imaging variables, and laboratory data, comparing between the relapsing and non-relapsing group. Qualitative variables were displayed by means of observed absolute value (n) and relative (%) frequencies. Quantitative variables were presented by their mean ± standard deviation, median, minimum, and maximum. Ordinal variables were presented by median, minimum, and maximum.

Subgroup comparisons were made using the Chi-square test for qualitative variables, applying the Fisher’s exact test. The Mann-Whitney U test and the Spearman’s Rank-Order Correlation test were employed to compare quantitative and ordinal variables. The receiver operating characteristic (ROC) curve analysis was performed to assess the accuracy of NLI and MLI in differentiating patients who experienced relapse or presented new T2-hyperintense lesions on MRI during the 1-year follow-up period.

Statistical significance was set at *p* < 0.05, with 95% confidence intervals (CIs). All analyses were conducted using IBM SPSS^®^ 27 software.

## 3 Results

### 3.1 Characterization of study population

From our sample cohort of pediatric MS patients, 15 (83.3%) were girls, and 3 (16.7%) were boys. The “relapse group” consisted of 3 (100%) girls, while 12 (80%) girls and 3 (20%) boys belonged to the “non-relapse group.” The mean diagnosis age ± standard deviation (SD) was 15.3 ± 2.1 years (range 13–17 years) in the relapse group, compared to 14.6 ± 3.2 years (range 6–17 years) in the non-relapse group. The diagnosis age was not significantly different between these groups (*p* = 0.912).

All patients had the relapsing-remitting (RRMS) phenotype of the disease. A DMT was initiated in 15 (83.3%) patients. Natalizumab was used in 5 (27.8%) patients, Interferon Beta-1a in 3 (16.7%), Glatiramer Acetate in 2 (11.1%), Interferon Beta-1b in 1 (5.6%), and Peginterferon Beta-1a in 1 (5.6%) patient. Three patients (16.7%) were included in clinical trials. In the relapse group, the prescribed DMT at the time of diagnosis was different for each patient: Interferon Beta-1a (33.3%), Natalizumab (33.3%) and Peginterferon Beta-1a (33.3%). There was no difference in the use of any DMT between the two groups.

The mean value ± SD of NLI was 5.17 ± 5.85 (range 1.57–11.92) and that of MLI was 0.35 ± 0.22 (range 0.19–0.59) in the relapse group. In the non-relapse group, the mean value ± SD of NLI was 2.19 ± 1.63 (range 1.12–7.32) and that of MLI was 0.24 ± 0.09 (range 0.14–0.44). There was no significant difference in the value of NLI or MLI between both groups. The relapse group had 2 (66.7%) patients with high value (which was defined above the median for the entire sample) of NLI (>1.89), and 2 (66.7%) patients with high value of MLI (>0.21), with one of these patients simultaneously having both biomarkers increased. There were not significantly more patients with higher NLI or MLI values in the relapse group than those in the non-relapse group.

The demographic characteristics of patients (age at the time of the diagnosis, sex), clinical data (MS phenotype classification, prescribed DMT at the time of diagnosis, and laboratory data), NLI and MLI closest to the first record point, according to belonging to the relapse and non-relapse groups, are summarized in Table 1.

**TABLE 1:** Characterization of study population.

	Total *n* = 18	Relapse in 1 year *n* = 3 (16,7%)	Non-relapse *n* = 15 (83,3%)	*p*
Sex	Female *n* = 15 (83.3%)	3 (100%)	12 (80%)	1.00
	Male *n* = 3 (16.7%)	0 (0%)	3 (20%)	
Age	Mean ± SD (Median; Min; Max)	15.3 ± 2.1 (16; 13; 17)	14.6 ± 3.2 (16; 6; 17)	0.912
Phenotype classification	RRMS *n* = 18 (100%)	3 (100%)	15 (100%)	
DMT	No treatment *n* = 3 (16.7%)	0 (0%)	3 (20%)	1.00
	Interferon Beta-1a *n* = 3 (16.7%)	1 (33.3%)	2 (13.3%)	0.442
	Interferon Beta-1b *n* = 1 (5.6%)	0 (0%)	1 (6.7%)	1.00
	Natalizumab *n* = 5 (27.8%)	1 (33.3%)	4 (26.7%)	1.00
	Glatiramer Acetate *n* = 2 (11.1%)	0 (0%)	2 (13.3%)	1.00
	Peginterferon Beta-1a *n* = 1 (5.6%)	1 (33.3%)	0 (0%)	0.167
	Clinical Trial *n* = 3 (16.7%)	0 (0%)	3 (20%)	1.00
NLI	Mean ± SD (Median; Min; Max)	5.17 ± 5.85 (2.02; 1.57; 11.92)	2.19 ± 1.63 (1.89; 1.12; 7.32)	0.498
	Increased *n* = 9 (50%)	2 (66.7%)	7 (46.7%)	1.00
MLI	Mean ± SD (Median; Min; Max)	0.35 ± 0.22 (0.25; 0.19; 0.59)	0.24 ± 0.09 (0.21; 0.14; 0.44)	0.360
	Increased *n* = 9 (50%)	2 (66.7%)	7 (46.7%)	1.00

DMT, disease-modifying therapies; Max, maximum value; Min, minimum value; MLI, monocyte/lymphocyte index; NLI, neutrophil/lymphocyte index; RRMS, relapsing-remitting MS; SD, standard deviation.

Clinical and imaging variables were described both at the time of diagnosis (number of previous relapses, the topography of MS lesions from previous relapses, EDSS score, the number of T2-hyperintense lesions and gadolinium-enhancing lesions on MRI, and the fulfillment of the revised 2010 McDonald diagnostic criteria), and after 12 months (EDSS score, T2-hyperintense lesions and gadolinium-enhancing lesions on MRI and the achievement of NEDA-3 state) (Table 2). The mean number of relapses prior to MS diagnosis ± SD was 1.00 ± 0.00 (range 1-1) in the relapse group and 1.20 ± 0.41 (range 1–2) in the non-relapse group. Based on the topographical involvement of the disease, spinal cord lesions were registered in 7 (38.9%) patients, optic nerve in 5 (27.8%), brainstem lesions in 3 (16.7%), and both optic nerve and brainstem in 3 (16.7%) patients. In the relapse group, the topography of MS lesions from previous relapses was different for each patient – spinal cord (33.3%), optic nerve (33.3%) and both optic nerve and brainstem (33.3%). The mean EDSS score ± SD at the first record point was 1.83 ± 0.76 (range 1.00–2.50) in the relapse group and 1.77 ± 0.90 (range 1.00–4.00) in the non-relapse group. After the 1-year follow-up period, the mean EDSS score ± SD was 1.33 ± 0.58 (range 1.00–2.00) in the relapse group and 1.20 ± 0.98 (range 0.00–3.50) in the non-relapse group. None of the patients demonstrated disability progression on the EDSS score.

**TABLE 2:** Clinical and imaging variables at the time of diagnosis and 1 year after.

	Total *n* = 18	Relapse in 1 year *n* = 3 (16,67%)	Non-relapse n = 15 (83,33%)	*p*
**At the time of the diagnosis:**				
Number of previous relapses	Mean ± SD (Median; Min; Max)	1.00 ± 0.00 (1; 1; 1)	1.20 ± 0.41 (1; 1; 2)	0.654
Topographical classification of previous relapses	Spinal Cord *n* = 7 (38.9%)	1 (33.3%)	6 (40%)	1.00
	Brainstem *n* = 3 (16.7%)	0 (0%)	3 (20%)	1.00
	Optic Nerve *n* = 5 (27.8%)	1 (33.3%)	4 (26.7%)	0.559
	Optic Nerve + Brainstem *n* = 3 (16.7%)	1 (33.3%)	2 (13.3%)	0.442
EDSS score	Mean ± SD (Median; Min; Max)	1.83 ± 0.76 (2.00; 1.00; 2.50)	1.77 ± 0.90 (1.50; 1.00; 4.00)	0.738
Number of T2-hyperintense lesions on MRI	(Median; Min; Max)	(≥10; ≥ 10; ≥ 10)	(≥10; 0–4; ≥ 10)	0.426
Number of Gd+ lesions on MRI	(Median; Min; Max)	(1–2; 0; ≥ 5)	(1–2; 0; ≥ 5)	1.00
2010 McDonald diagnostic criteria	No Criteria *n* = 2 (11.1%)	1 (33.3%)	1 (6.7%)	0.314
	DIS *n* = 2 (11.1%)	0 (0%)	2 (13.3%)	0.442
	DIT *n* = 1 (5.6%)	0 (0%)	1 (6.7%)	1.00
	DIS + DIT *n* = 13 (72.2%)	2 (66.7%)	11 (73.3%)	1.00
**12 months after the diagnosis:**				
EDSS score	Mean ± SD (Median; Min; Max)	1.33 ± 0.58 (1.00; 1.00; 2.00)	1.20 ± 0.98 (1.00; 0.00; 3.50)	0.824
	Disability progression *n* = 0 (0%)	0 (0%)	0 (0%)	
T2-hyperintense lesions on MRI	Presence *n* = 11 (61.1%)	3 (100%)	8 (53.3%)	0.245
	Absence *n* = 7 (38.9%)	0 (0%)	7 (46.7%)	
Number of T2-hyperintense lesions on MRI	(Median; Min; Max)	(5–9; 1–4; ≥ 10)	(1–4; 0; ≥ 10)	0.301
Gd+ lesions on MRI	Presence *n* = 5 (27.8%)	1 (33.3%)	4 (26.7%)	1.00
	Absence *n* = 13 (72.2%)	2 (66.7%)	11 (73.3%)	
Number of Gd+ lesions on MRI	Mean ± SD (Median; Min; Max)	1,00 ± 1,73 (0; 0; 3)	0.53 ± 1.06 (0; 0; 3)	0.824
NEDA-3 state	Achieved *n* = 6 (33.3%)	0 (0%)	6 (40%)	0.515
	Not achieved *n* = 12 (66.7%)	3 (100%)	9 (60%)	

DIS, dissemination in space; DIT, dissemination in time; EDSS, expanded disability status scale; Gd+ , gadolinium-enhancing; Max, maximum value; Min, minimum value; NEDA, no evidence of disease activity; SD, standard deviation.

At the time of diagnosis, both groups had, as a median, ≥ 10 T2 hyperintense lesions on MRI, as well as 1–2 lesions (also as a median), with enhancement after gadolinium administration. In the following year, there were 11 (61.1%) patients with new T2-hyperintense lesions on MRI, including all those belonging to the relapse group, and 5 (27.8%) patients with gadolinium-enhancing lesions on MRI, of whom only 1 (33.3%) belonged to the relapse group. At this timepoint, the median number of T2-hyperintense lesions on MRI was 5–9 in the relapse group and 1–4 in the non-relapse group. The mean number of gadolinium-enhancing lesions on MRI ± SD was 1.00 ± 1.73 (range 0–3) and 0.53 ± 1.06 (range 0–3), correspondingly. Dissemination in space (DIS) and dissemination in time (DIT) criteria were found concurrently in 13 (72.2%) patients at the time of diagnosis, of whom 2 (66.7%) relate to the relapse group. The NEDA-3 state was not achieved by 12 (66.7%) MS pediatric patients, which corresponds to the entire relapse group, together with 9 (60%) patients of the non-relapse group, due to MRI activity of the disease.

### 3.2 Association of NLI and MLI with other variables

The mean value ± SD of NLI was 2.94 ± 2.95 (range 0.12–11.92) and that of MLI was 0.27 ± 0.13 (range 0.14–0.59) within the female population. For the male population, the mean value ± SD of NLI was 1.40 ± 0.11 (range 1.33–1.52) and that of MLI was 0.22 ± 0.05 (range 0.18–0.28). For both NLI (*p* = 0.100) and MLI (*p* = 0.824), there was no statistically significant difference in the mean value between girls and boys. In the subsequent year after diagnosis, patients who presented new T2-hyperintense lesions on MRI had a mean NLI value ± SD of 3.23 ± 3.37 (range 0.84–11.92), compared to 1.83 ± 1.00 (range 0.12–3.09) in patients without new T2-hyperintense lesions on MRI. The mean MLI value ± SD was 0.27 ± 0.13 (range 0.14–0.59) in patients with new T2-hyperintense lesions on MRI, while it was 0.24 ± 0.09 (range 0.15–0.41) in those without new T2-hyperintense lesions. NLI and MLI had mean values ± SD of 1.43 ± 0.39 (range 0.84–1.90) and 0.22 ± 0.04 (range 0.18–0.28), correspondingly, in patients with new gadolinium-enhancing lesions on MRI. Alternately, in those without new gadolinium-enhancing lesions on MRI, the mean value ± SD was 3.17 ± 3.12 (range 0.12–11.92) for NLI and 0.27 ± 0.13 (range 0.14–0.60) for MLI. The mean NLI value was not statistically different between patients with or without T2-hyperintense lesions on MRI (*p* = 0.659), although statistical significance was noted for patients with or without gadolinium-enhancing lesions on MRI (*p* = 0.046). Regarding MLI, the mean value was not statistically different between patients with or without T2-hyperintense (*p* = 0.596) or gadolinium-enhancing lesions (*p* = 0.849) on MRI.

Pediatric-onset MS (POMS) patients who achieved NEDA-3 state had a mean value ± SD of NLI of 1.82 ± 1.09 (range 0.12–3.09), and that of MLI was 0.24 ± 0.10 (range 0.15–0.41). Those who did not achieve NEDA-3 state had a mean value ± SD of 3.12 ± 3.24 (range 0.84–11.92) and 0.27 ± 0.13 (range 0.14–0.60), for NLI and MLI, respectively. For both biomarkers, the mean value did not present a statistically significant difference between patients who achieved NEDA-3 state, in comparison with patients who did not achieve it (*p* = 0.682, for NLI; *p* = 0.616, for MLI).

None of the biomarkers (NLI and MLI), showed statistically significant correlations with either clinical or imaging variables assessed within our sample cohort – sex, age at the time of the diagnosis; after the 1-year follow-up period – EDSS score, presence of T2-hyperintense lesions and gadolinium-enhancing lesions on MRI, number of new T2-hyperintense lesions and gadolinium-enhancing lesions, and fulfilment of the NEDA-3 state. Table 3 outlines the correlations between NLI and MLI in the studied population.

**TABLE 3:** Correlations between NLI and MLI and variables of interest in POMS patients.

		NLI		MLI	
	**Total** ***n* = 18**	**Mean ± SD** **(Median; Min; Max)**	***p***	**Mean ± SD** **(Median; Min; Max)**	***p***
Sex	Female *n* = 15 (83.3%)	2.94 ± 2.95 (2.17; 0.12; 11.92)	0.100	0.27 ± 0.13 (0.24; 0.14; 0.59)	0.824
	Male *n* = 3 (16.7%)	1.40 ± 0.11 (1.34; 1.33; 1.52)		0.22 ± 0.05 (0.19; 0.18; 0.28)	
Age			0.549 (*r* = 0,151)		0.411 (*r* = 0,206)
**12 months after the diagnosis:**					
EDSS score			0.185 (*r* = 0,327)		0.401 (*r* = 0,211)
T2-hyperintense lesions on MRI	Presence *n* = 11 (61.1%)	3.23 ± 3.37 (2.02; 0.84; 11.92)	0.659	0.27 ± 0.13 (0.25; 0.14; 0.59)	0.596 0.258 (*r* = 0,281)
	Absence *n* = 7 (38.9%)	1.83 ± 1.00 (1.89; 0.12; 3.09)		0.24 ± 0.09 (0.21; 0.15; 0.41)	
	No		0.983 (*r* = −0,005)		
Gd+ lesions on MRI	Presence *n* = 5 (27.8%)	1.43 ± 0.39 (1.52; 0.84; 1.90)	0.046	0.22 ± 0.04 (0.21; 0.18; 0.28)	0.849
	Absence *n* = 13 (72.2%)	3.17 ± 3.12 (2.17; 0.12; 11.92)		0.27 ± 0.13 (0.24; 0.14; 0.60)	
NEDA-3 state	Achieved *n* = 6 (33.3%)	1.82 ± 1.09 (1.92; 0.12; 3.09)	0.682	0.24 ± 0.10 (0.21; 0.15; 0.41)	0.616
	Not achieved *n* = 12 (66.7%)	3.12 ± 3.24 (1.96; 0.84; 11.92)		0.27 ± 0.13 (0.23; 0.14; 0.60)	

EDSS, expanded disability status scale; Gd+ , gadolinium-enhancing; Max, maximum value; Min, minimum value; NEDA, no evidence of disease activity; SD, standard deviation.

### 3.3 Ability of NLI and MLI to differentiate patients with evidence of relapse, new T2-hyperintense or gadolinium-enhancing lesions on MRI after 1 year of follow-up

**FIGURE 2:**
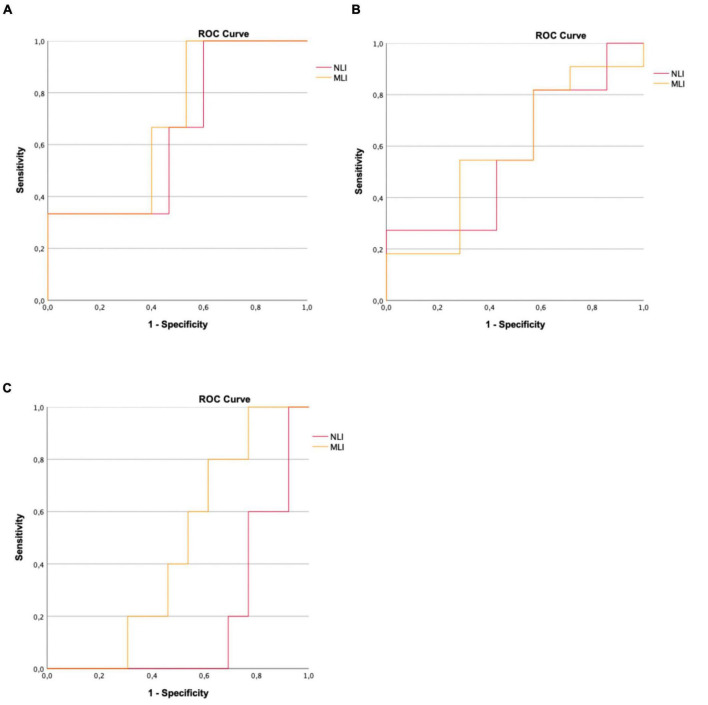
**(A)** ROC curve for NLI and MLI to assess accuracy when differentiating patients who experienced relapse during the 1-year follow-up period. **(B)** ROC curve for NLI and MLI to assess accuracy when differentiating patients who presented new T2-hyperintense lesions on follow-up MRI. **(C)** ROC curve for NLI and MLI to assess accuracy when differentiating patients who presented new gadolinium-enhancing lesions on follow-up MRI.

We performed ROC analysis to evaluate the ability of NLI and MLI to discriminate between the relapse group and the non-relapse group. This ROC curve can be seen in Figure 2A. We obtained an area under the curve (AUC) of 0.644 (*p* = 0.441) for NLI and 0.689 (*p* = 0.314) for MLI. In addition, to evaluate the ability of NLI and MLI to distinguish between patients with and without new T2-hyperintense lesions on follow-up MRI, we conducted a second ROC analysis, which curve is displayed in Figure 2B. The AUC was 0.571 (*p* = 0.618) for NLI and 0.584 (*p* = 0.556) for MLI. Finally, to assess the potential of NLI and MLI to differentiate between patients with and without new gadolinium-enhancing lesions on follow-up MRI, we carried out another ROC analysis, which curve is shown in Figure 2C. The AUC was 0.185 (*p* = 0.043) for NLI and 0.462 (*p* = 0.805) for MLI.

## 4 Discussion

We described a cohort of 18 patients diagnosed with POMS, 3 of whom registered occurrence of relapses in the first year after diagnosis, trying to evaluate whether the NLI and MLI could correlate with some clinical and imaging variables of interest, 1 year after the diagnosis of POMS and after the implementation of the respective therapeutic strategy. We included 15 girls and 3 boys, and the relapse group consisted only of 3 (100%) girls, with a mean age of 15.3 years. The non-relapse group consisted of 12 (80%) girls and 3 (20%) boys, and the mean age was 14.6 years. Mean age at diagnosis and gender of participants were not significantly different between groups and this finding is consistent with previous studies, that have also not found any significant association between sex, age, and clinical outcome measures, as the number of relapses (Banwell et al., 2007; Renoux et al., 2007).

All patients had a RRMS phenotype, which also agrees with literature (Banwell et al., 2007; Renoux et al., 2007). This aligns with a previous monocentric Portuguese study that found a great uniformity of the clinical phenotype below 21 years of age (Silva and Sá, 1999). We noticed that, regarding disability and disease progression, the EDSS score was lower after the first year, compared to that registered at the time of diagnosis. This applied to both those who had experienced relapses and those who had not, although the mean EDSS scores were higher in the relapse-group. The fact that none of the patients in this study showed worsening of disability may be due to the effectiveness of the immunomodulatory treatments, which were initiated earlier in this population (Pohl et al., 2007), as well as the timing of the first EDSS assessment, which was frequently carried out during a relapse, at the moment of diagnosis. It is possible that this fact may be biased by the still relatively short follow-up time in the cohort of patients recruited for the study. Typically, pediatric MS patients who experience a relapse are expected to fully recover within a year, without presenting cumulative disability (Narula, 2016). While some previous studies have indicated that most children with RRMS maintain a mild motor disability (Peña et al., 2006), other have emphasized the unpredictable course of POMS, with reports of significant and gradual progression (Boiko et al., 2002).

A DMT was initiated in 15 (83.3%) patients, while 3 (16.7%) remained treatment naïve. Natalizumab was the first-line treatment in most patients (27.8%), followed by the longer in use Interferons (Interferon Beta-1a, 16.7%; Interferon Beta-1b, 5.6%) and Glatiramer acetate (11.1%). The data currently available for POMS patients who did not respond to initial immunomodulatory treatments indicate that Natalizumab is effective in reducing clinical relapses in most cases (Huppke et al., 2008; Arnal-Garcia et al., 2013). It is important to note that the vast majority of patients included in the study started their treatment before the formal approval of Fingolimod, Teriflunomide and Dimethylfumarate for the treatment of POMS. During the 1-year follow-up period, only 1 (20%) patient who was being treated with Natalizumab experienced a relapse. All patients from the relapse group were prescribed a DMT at the diagnosis, although being different for each patient: Natalizumab (33.3%), Interferon Beta-1a (33.3%), and Peginterferon Beta-1a (33.3%). We did not identify a statistically significant difference in any DMT use between the two groups. Still, it was previously described that there were significantly more patients who were prescribed Interferon Beta-1b in the relapse group (6.06%) than in the non-relapse group (1.38%) (Huang et al., 2022). Many studies have shown a decline in relapse rate after starting first-line treatments (Chitnis et al., 2012). Despite this, some children still experience breakthrough disease, whose definition, proposed by the International Pediatric Multiple Sclerosis Study Group (IPMSSG), may involve an increase or no reduction in relapse rate, or ≥ 2 clinical relapses within 12 months (Macaron et al., 2019).

In our study, we collected data on imaging variables at two distinct evaluation periods: at the time of diagnosis and 12 months later. There was no statistically significant difference between the relapse group and the non-relapse group at both registration points, in terms of median number of T2-hyperintense or gadolinium-enhancing lesions on MRI. Previous research has shown that both a high relapse rate and a high T2-hyperintense lesion load can predict future disability, therefore being routinely used for prognostic purposes (Fisniku et al., 2008; Jokubaitis et al., 2016). However, numerous studies have revealed a limited correlation between clinical and imaging outcome measures in terms of MS disease progression, which validates the concept of clinico-radiological dissociation of disease activity (Healy et al., 2017), which is particularly relevant in POMS.

To evaluate the usefulness of the NLI and MLI biomarkers, the following results were obtained: The relapse group had higher mean, minimum, and maximum values for both biomarkers (5.17 ± 5.85, ranging from 1.57 to 11.92 for NLI, and 0.35 ± 0.22, ranging from 0.19 to 0.59 for MLI), in comparison to the non-relapse group (2.19 ± 1.63, ranging from 1.12 to 7.32 for NLI, and 0.24 ± 0.09, ranging from 0.14 to 0.44 for MLI). Olsson et al. (2021) conducted a review of various studies and found that patients with active MS generally have a median NLI range of 2.5–4.89, while those in a remitting stage of the disease typically have a range of 1.61–2.22. This is in line with our study’s findings. Moreover, the relapse group had more patients with higher NLI value (NLI > 1.89, 66.7%) than those in the non-relapse group (46.7%). The relapse group also had a higher percentage of patients with increased MLI value (MLI > 0.21, 66.7%) than those in the non-relapse group (46.7%). The mean, minimum, and maximum values of both biomarkers were found to be higher (3.23 ± 3.37, ranging from 0.84 to 11.92 for NLI, and 0.27 ± 0.13, ranging from 0.14 to 0.59 for MLI) in the group of patients who exhibited new T2-hyperintense lesions on MRI, after 1 year of follow-up. In contrast, in the group of patients who did not accumulate new T2-hyperintense lesions on MRI, the values were 1.83 ± 1.00 (range 0.12–3.09) for NLI and 0.24 ± 0.09 (range 0.15–0.41) for MLI. Similarly, patients who did not achieve NEDA-3 state exhibited higher values for both ratios. NLI and MLI use the same calculation method, which involves a ratio of lymphocytes, and both biomarkers are based on components of WBC (neutrophils and monocytes). It is possible that these two ratios change in a similar way due to shared factors that influence them.

In the ROC analysis performed, in which an attempt was made to explore the discriminatory potential of the indices (ability to differentiate patients with evidence of relapse, new T2-hyperintense or gadolinium-enhancing lesions on MRI after 1 year of follow-up) it was not possible to obtain any result with statistical significance. We believe it is difficult to draw anything clinically robust from this conclusion, at this stage. The protocol proved to be feasible and simple to apply and it will now be necessary to increase the sample size to deepen the discriminatory potential of these indices.

Nevertheless, despite the lack of statistically significant results, it is worth mentioning the pattern observed. It is interesting to consider the factors that could have influenced this result, with one conceivable explanation being the prolonged time gap between conducting the blood test and the occurrence of relapse. In our cohort, we were unable to validate the reliability of NLI and MLI biomarkers as predictors of the occurrence of relapses at 1 year after the diagnosis, disability progression, as measured by the EDSS, and lesion accumulation on MRI. In a 3-year follow-up study conducted by Yetkin and Mirza (2020), no correlation was found between NLI and risk of MS relapse. On the other hand, Hemond et al. (2019) showed that elevated NLI and MLI values are highly indicative of worsened disability related to MS, regardless of demographics, clinical, and psychosocial factors. Scientific evidence has already been described regarding NLI as informative biomarker for predicting the prognosis of various diseases (Okyay et al., 2013; Sen et al., 2014; Akıl et al., 2015; Dirican et al., 2016; Erre et al., 2019; Lattanzi et al., 2019; Wang et al., 2019; Sayed et al., 2020), including MS (Olsson et al., 2021). As for MLI, the literature is not extensive regarding its role in MS course, although a correlation was found between this biomarker and other conditions (Du et al., 2017; Ren et al., 2017; Huang et al., 2018).

It should be noted that our study has several limitations. Firstly, as we conducted a retrospective observational study using data exclusively from the Hospital Pediátrico (Centro Hospitalar e Universitário de Coimbra) research database, it is limited by its unicentric and retrospective nature, which is susceptible to selection and information biases. The small number of pediatric MS patients included in this study (and it should be highlighted that the relapse group was defined only by 3 subjects) does not allow a robust association analysis for the different variables and generalization to other populations. Even though it is small, this group of patients is heterogeneous enough to have motivated very different choices of DMT, which corroborates the different clinical profile of the cases, even at the time of their diagnosis (and this heterogeneous prescription proved to be effective, given the lack of progression of disability in our population and the low number of patients with a relapse in the time period considered). Consequently, despite observing some trends, firm conclusions cannot, for now, be drawn. Second, we were unable to analyze the influence of different DMT on both NLI and MLI, as there was not enough time elapsed for this evaluation. The DMT was prescribed at the time of diagnosis, just as the CBC was performed during that same period, without any subsequent record being made. Third, since there is no established cut-off for NLI and MLI values in MS patients, we considered NLI and MLI to be increased for values above the median in our pediatric sample cohort, as it was done in a previous study. Nonetheless, according to this dichotomy, we acknowledged that there may be patients whose NLI or MLI values were considered low, although it can include normal and abnormal ranges, with different relapse rates. Fourth, even though the focus was on a situation that reflects clinical routine practice, we consider it appropriate to recognize that while the complete differential blood count can provide an overall measure of the immune function, it has limitations in its ability to distinguish between different lymphocyte subsets, such as Th1/Th17, that play an important role in the pathogenesis of the disease (van Langelaar et al., 2018).

Future research should focus on larger, multicentric patient populations and well-controlled prospective studies to determine the accuracy and predictive value of NLI or MLI as biomarkers for MS relapse, disability progression, or MRI lesion load accumulation, also in pediatric environment. It is also needed to clarify how different DMT affect NLI and MLI, as we know that these biomarkers could be useful in decision-making processes for therapeutic approach, to “treat-to-target.” Some studies reported how DMT can manipulate the neutrophil count or function, which reinforces the relevance of these cells in MS pathophysiology. The protocol we used proved to be feasible, and we intend to replicate it in a multicenter study, in the near future.

## 5 Conclusion

Neutrophil-lymphocyte index and MLI characterize the balance between neutrophil, monocytes, and lymphocyte levels, respectively. These ratios can be quickly and cheaply obtained from the CBC that is routinely performed on patients, at the time of diagnosis. The simplicity on acquiring this laboratory data makes them useful biomarkers as a screening tool in longitudinal follow-up patients. Although the exact values above which we classify NLI and MLI as elevated are not yet established, we can track the patient’s inflammatory status by monitoring their relative NLI or MLI values over time. Given the inflammation relevance in an early stage of childhood-onset MS, the NLI and MLI may be predictive of the disease course or outcome, particularly, the risk of relapse, the risk of disability progression, as measured by the EDSS score, and the risk of lesion accumulation on MRI. Also, a crucial aim for future research is to elucidate the influence of different DMT on NLI and MLI, in early MS. The goal is to better apply the concept of “treat-to-target,” in which clinicians intend to provide the patient with a state of absence of MS activity, bearing in mind the positive impact on the quality of life of patients and their families, in the short, medium, and long term.

Multicentre and well-controlled prospective studies should be conducted to validate (or not) the pattern that we observed resulting from inferential statistical analyses, in a sample with a very limited size. Our study is intended to serve as the basis for a future project collecting data from multiple centers–by increasing the sample size and using the same protocol as our study, it is possible that data of additional clinical interest will be obtained, in a very special population, such as children and adolescents diagnosed with MS.

## Data availability statement

The original contributions presented in this study are included in the article/supplementary material, further inquiries can be directed to the corresponding author.

## Ethics statement

The studies involving humans were approved by the Local Ethics Committee (Centro Hospitalar e Universitário de Coimbra). The studies were conducted in accordance with the local legislation and institutional requirements. Written informed consent for participation in this study was provided by the participants’ legal guardians/next of kin.

## Author contributions

FP: Conceptualization, Data curation, Formal analysis, Investigation, Methodology, Project administration, Resources, Software, Validation, Visualization, Writing—original draft, Writing—review and editing. LG: Data curation, Formal analysis, Investigation, Software, Writing—original draft. AJ: Data curation, Formal analysis, Methodology, Writing—review and editing. MM: Formal analysis, Software, Supervision, Writing—review and editing. CS: Data curation, Writing—review and editing. JA: Data curation, Writing—review and editing. JR: Data curation, Writing—review and editing. CP: Data curation, Writing—review and editing. CR: Supervision, Writing—review and editing.
